# Association of ERCC1 rs3212986 & ERCC2 rs13181 polymorphisms with the risk of glioma

**DOI:** 10.12669/pjms.306.5221

**Published:** 2014

**Authors:** Qing-ke Cui, Jian-xin Zhu, Wei-dong Liu, Yun-hua Wang, Zhi-gang Wang

**Affiliations:** 1 Qing-ke Cui, Department of Neurosurgery, Liaocheng People's Hospital, Liaocheng Shandong, 252000, P. R. China; 2 Qing-ke Cui, Department of Neurosurgery, Liaocheng People's Hospital, Liaocheng Shandong, 252000, P. R. China; 3 Wei-dong Liu, Department of Neurosurgery, Liaocheng People's Hospital, Liaocheng Shandong, 252000, P. R. China; 4 Yun-hua Wang, Department of Neurosurgery, Liaocheng People's Hospital, Liaocheng Shandong, 252000, P. R. China; 5 Zhi-gang Wang, Department of Neurosurgery, Liaocheng People's Hospital, Liaocheng Shandong, 252000, P. R. China

**Keywords:** ERCC1, ERCC2, Polymorphism, Glioma

## Abstract

***Objective:*** ***:*** Several previous studies have reported the role variant of ERCC1 rs3212986 and ERCC2 rs13181 polymorphisms in the risk of glioma, but the results of these studies are inconsistent. Therefore, we aimed to conduct a meta-analysis to investigate the role of ERCC1 rs3212986 and ERCC2 rs13181 on the risk of glioma.

***Methods:*** A comprehensive research was conducted through the databases of Pubmed, EMBASE and the China National Knowledge Infrastructure (CNKI) platforms until June 1, 2014, including 14 eligible case-control studies.

***Results:*** Our meta-analysis found that ERCC1 rs3212986 AA genotype was significantly associated with increased risk of glioma compared with CC genotype, and the pooled OR (95%CI) was 1.29(1.07-1.55). By subgroup analysis, ERCC1 rs3212986 AA genotype was found to be significantly correlated with increased glioma risk in Chinese population (OR=1.37, 95%CI=1.07, 1.55), Similarly, we found that ERCC2 rs13181 GT and TT genotypes were significantly associated with increased risk of glioma in Chinese population, with ORs(95%CI) of 1.47(1.17-1.85) and 1.50(1.02-2.22). But ERCC1 rs3212986 and ERCC2 rs13181 polymorphisms had no significant association with glioma risk in Caucasian populations. By begg’s funnel plot, we found that no publication bias was existed in this meta-analysis.

***Conclusion: ***Our meta-analysis suggested that ERCC1 rs3212986 and ERCC2 rs13181 polymorphism play an important risk factor for brain tumor development in Chinese population, but no association in Caucasian populations.

## INTRODUCTION

Gliomas are the most common occurring type of brain tumors worldwide, accounting for approximately 80% of all malignant brain tumors.^1^ In 2000, it is estimated that the annual incidence of brain tumor was about 3.9/105 in men and 2.8 per 10^5^ in women in Chinese population, and it is about 2–3 per 100,000 in Europe and North America.^2^^-^^4^ The incidence of brain tumors has increased in recent several years, and the incidence shows significant different in terms of gender, age, race, ethnicity, and even geographical region.^5^^,^^6^ However, the real etiology of glioma is still not well understood. Previous studies reported that mutations of many genes can affect the risk of glioma.^7^^-^^9^

It is well known that changes in DNA induced by exposure to environmental and endogenous agents may result in genetic instability and mutagenesis. DNA repair mechanisms play an important role in maintaining genomic integrity and preventing carcinogenesis. Therefore, if genetic variations in DNA repair genes change the amount or activity of the proteins that they encode, they might also increase the risk of disease and the risk of cancer in particular. Excision repair cross complementation group 1 (ERCC1) and ERCC2 lie on chromosome 19q13.3, and they play an important role in NER pathway.^10^

Several previous studies have reported the role variant of ERCC1 rs3212986 and ERCC2 rs13181 polymorphisms in the risk of glioma, but the results of these studies are inconsistent. Therefore, we aimed to conduct a meta-analysis to investigate the role of ERCC1 rs3212986 and ERCC2 rs13181 on the risk of glioma.

## METHODS

***Search strategy*** ***:*** A comprehensive research was conducted through the databases of Pubmed, EMBASE and the China National Knowledge Infrastructure (CNKI) platforms until June 1, 2014. The literature search was conducted using the following terms: “glioma” or “brain tumor”, “polymorphism” or “variant”, and “ERCC1 C8092A”, “ERCC2 Lys751Gln”, “rs3212986” or “rs13181”. The reference lists of articles included for review and past meta-analyses were examined for any further relevant publications. No publication date or language restrictions were applied.

***Study selection*** ***:*** The inclusion criteria for studies were as follows: studies are case-control design; studies evaluated the association between ERCC1 rs3212986 and ERCC2 rs13181 polymorphisms and glioma risk; studies reported the results of available genotype frequencies.

The exclusion criteria for studies were as follows: articles only have an abstract, review articles and comments; studies are overlapped with other studies; studies have no comparison or control group; studies have no data of genotype frequencies.

Corresponding authors were contacted in an attempt to obtain unreported genotype counts if studies were otherwise eligible.

**Data extraction**

Two authors independently screened the electric search with all terms. Duplications and obviously irrelevant studies were excluded according to the exclusion criteria. We extracted the full texts of all eligible studies according to the inclusion criteria. The study ID, study design, participation number, case-control number and genotype information were extracted for each eligible study.

**Statistical analysis**

All meta-analysis analysis was conducted by STATA 9.0 software. A chi-square (χ^2^) was taken to evaluate the Hardy–Weinberg equilibriums in control groups. The pooled odds ratios (OR) and 95% confidence interval (CI) were taken to calculate the role of ERCC1 rs3212986 and ERCC2 rs13181 polymorphisms on the risk of glioma. The heterogeneity between studies was estimated by *I*^2^ test and heterogeneity Q statistic test. When *I*^2^ were at the range of 0-25%, there was no degree of heterogeneity. When *I*^2^ were at the range of 25-50%, there was moderate heterogeneity. When *I*^2^ were at the range of 75-100%, there was great heterogeneity. A random-effects model or fixed-effect model was taken to calculate the pooled OR (95%CI) according to the degree of heterogeneity between studies. The publication bias in studies was calculated using Begg’s funnel plot and Egger’s test.

## RESULTS

***Characteristics of eligible publications: ***Our comprehensive literature search identified a total of 57 studies for ERCC1 rs3212986 and ERCC2 rs13181 based on their titles. Finally, 14 eligible case-control studies were selected, including eight studies for ERCC1 C8092A and 11 studies for ERCC2 Lys751Gln.^11^^-^^24^

Eight studies reported the association between ERCC1 rs3212986 polymorphism and glioma risk, including 3008 glioma cases and 4319 controls (Table-I).^11^^-^^18^ 11 studies reported the association between ERCC2 Lys751Gln polymorphism and glioma risk, including 3456 glioma cases and 4957 controls (Table-II).^12^^-^^15^^,^^19^^-^^25^ Six studies were conducted in Chinese population, and the other seven studies were conducted in Caucasian populations. 

The genetic distributions of ERCC1 rs3212986 in eight studies and ERCC2 rs13181 in nine studies were in according to Hardy-Weinberg Equilibrium (HWE). Only one study about ERCC2 rs13181 deviated from Hardy-Weinberg equilibrium in the control groups. 

There was no significant heterogeneity between pooled studies, and thus we performed a fixed-effect model to assess the association between ERCC1 rs3212986 and ERCC2 rs13181 and risk of glioma. Our meta-analysis found that ERCC1 8092 AA genotype was significantly associated with increased risk of glioma compared with CC genotype, and the pooled OR (95%CI) was 1.29(1.07-1.55). However, we did not find significant association between ERCC2 rs13181 polymorphisms and risk of glioma.

By subgroup analysis, ERCC1 rs3212986 AA genotype was found to be significantly correlated with increased glioma risk in Chinese population (OR=1.37, 95%CI=1.07, 1.55) (Figure-I). Similarly, we found that ERCC2 rs13181 GT and TT genotypes were significantly associated with increased risk of glioma in Chinese population, with ORs(95%CI) of 1.47(1.17-1.85) and 1.50(1.02-2.22). But ERCC1 rs3212986 and ERCC2 rs13181 polymorphisms had no significant association with glioma risk in Caucasian populations (Figure II and Figure III). 

By begg’s funnel plot, we found that the shapes of the funnel plots for ERCC1 rs3212986 and ERCC2 rs13181 were symmetry, which suggests no publication bias in this meta-analysis (Figure IV and Figure V). Moreover, sensitivity analysis showed that the pooled OR did not alter after excluding one largest sample size study.

**Figure I:**
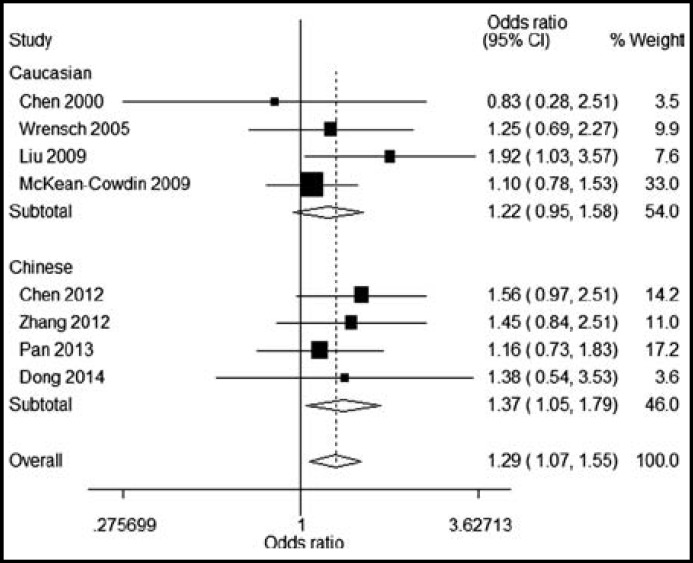
Subgroup analysis for the role of ERCC1 rs3212986 AA on the glioma risk

**Figure-II:**
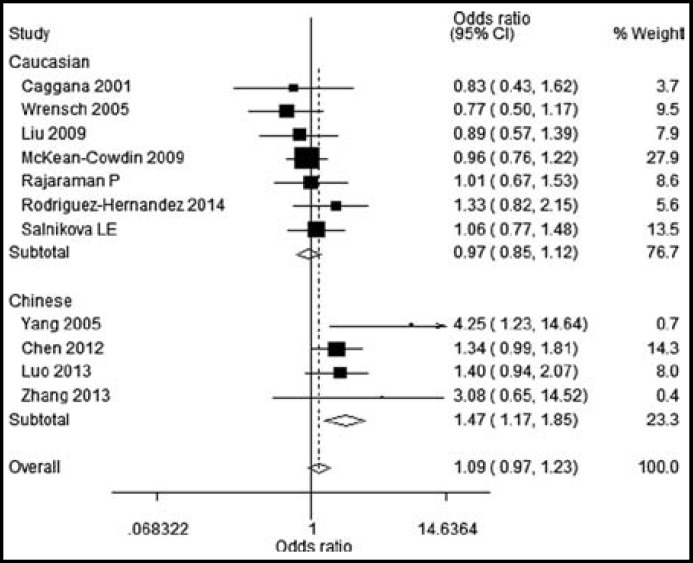
Subgroup analysis for the role of ERCC2 rs13181 GT on the glioma risk

**Figure III:**
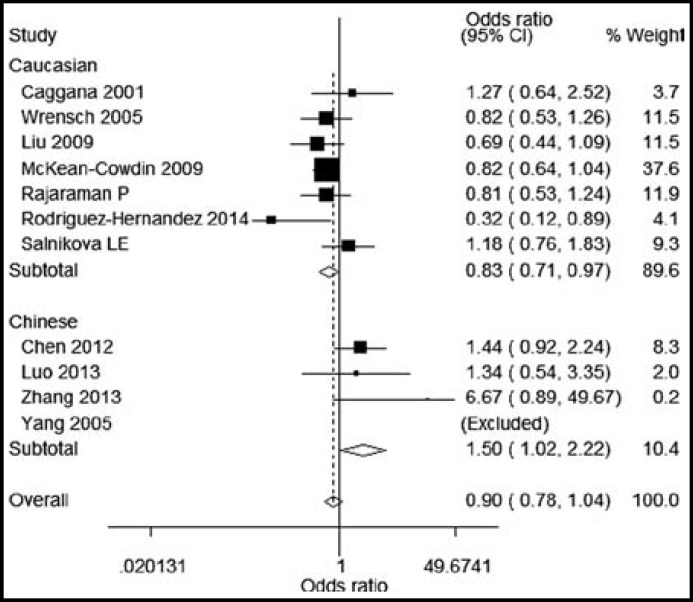
Subgroup analysis for the role of ERCC2 rs13181 TT on the glioma risk

**Figure-IV:**
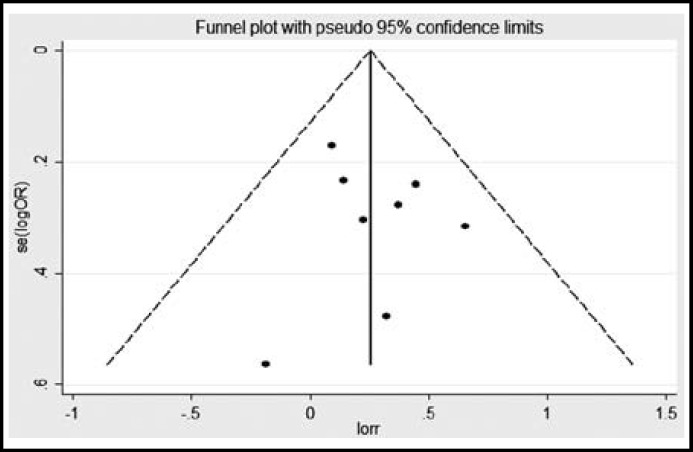
Funnel plots of the association between ERCC1 rs3212986 polymorphism and risk of glioma

**Figure-V:**
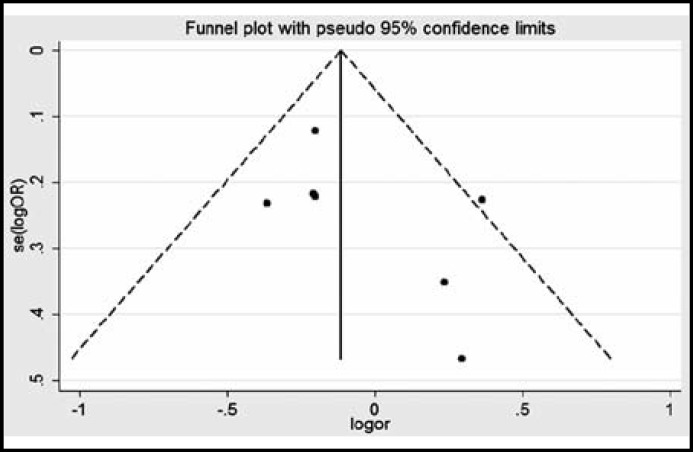
Funnel plots of the association between ERCC2 rs13181 polymorphism and risk of glioma

## DISCUSSION

To the best of our knowledge, the current meta-analysis comprehensively analyze the association between ERCC1 rs3212986 and ERCC2 rs13181 polymorphism and glioma risk. The results of this meta-analysis suggest that the ERCC1 rs3212986 and ERCC2 rs13181 polymorphism polymorphisms was significantly associated with increased risk of glioma in Chinese population, but no association in Caucasian Chinese.

**Table-I:** Meta-analysis of the association of ERCC1 rs3212986 polymorphism with the glioma risk

Study ID		Country	Cases			Total cases	Controls			Total controls	P for HWE	OR(95%CI)^1^	
			CC	CA	AA		CC	CA	AA			CA vs CC	AA vs CC
Chen P	2000	Caucasian	73	43	6	122	81	70	8	159	0.47	0.68(0.42-1.12)	0.83(0.28-2.51)
Wrensch M	2005	Caucasian	206	144	25	393	237	184	23	410	0.09	0.90(0.68-1.20)	1.25(0.69-2.27)
Liu Y	2009	Caucasian	208	130	31	72	219	126	17	302	0.83	1.09(0.80-1.48)	1.92(1.03-3.57)
McKean-Cowdin R	2009	Caucasian	557	361	59	369	1087	728	105	362	0.24	0.97(0.82-1.14)	1.10(0.78-1.53)
Chen DQ	2012	Chinese	202	141	50	977	221	154	35	1920	0.28	1.00(0.74-1.35)	1.56(0.97-2.51)
Zhang N	2012	Chinese	123	98	36	443	144	105	29	444	0.14	1.09(0.76-1.58)	1.45(0.84-2.51)
Pan WR	2013	Chinese	229	169	45	375	241	162	41	444	0.08	0.10(0.83-1.45)	1.16(0.73-1.83)
Dong YS	2014	Chinese	33	32	7	257	137	144	21	278	0.94	0.92(0.54-1.58)	1.38(0.54-3.53)
Pooled results			1631	1118	259	3008	2367	1673	279	4319		0.98(0.89-1.09)	1.29(1.07-1.55)
P for heterogeneity^a^												0.793	0.762
*I* ^2^ test												0%	0%

OR: odds ratio; CI: confidence interval; HWE: Hardy–Weinberg equilibrium. ^1^*P *< 0.05, it was considered statistically significant.

**Table-II:** Meta-analysis of the association of ERCC2 rs13181 with the glioma risk

Study ID		Ethnicity	Cases			Total	Controls			Total	P for HWE	OR(95%CI)^1^	
			GG	GT	TT		GG	GT	TT			CT vs GG	TT vs GG
Caggana M	2001	Caucasian	23	63	62	148	23	76	49	148	0.46	0.83(0.43,1.62)	1.27(0.64,2.52)
Wrensch M	2005	Caucasian	57	169	139	365	55	213	164	432	0.27	0.77(0.50,1.17)	0.82(0.53,1.26)
Liu Y	2009	Caucasian	56	172	139	367	45	156	161	362	0.45	0.89(0.57,1.39)	0.69(0.44,1.09)
McKean-Cowdin R	2009	Caucasian	143	480	376	989	256	891	823	1970	0.54	0.96(0.76,1.22)	0.82(0.64,1.04)
Rajaraman P	2010	Caucasian	52	171	128	351	66	215	200	481	0.5	1.01(0.67,1.53)	0.81(0.53,1.24)
Salnikova LE	2013	Caucasian	100	135	49	284	171	217	71	459	0.87	1.06(0.76-1.50)	1.18(0.74-1.87)
Rodriguez-Hernandez I	2014	Caucasian	51	59	5	115	92	80	28	200	0.13	1.33(0.80-2.21)	0.32(0.09-0.92)
Yang D	2005	Chinese	103	32	0	135	41	3	0	44	0.81	4.24(1.21-22.71)	-
Chen DQ	2012	Chinese	139	198	56	393	175	186	49	410	0.97	1.34(0.99,1.81)	1.44(0.92,2.24)
Luo KQ	2013	Chinese	230	58	9	297	343	62	10	415	0.03	1.40(0.94,2.07)	1.34(0.54,3.35)
Zhang X	2013	Chinese	3	6	3	12	20	13	3	36	0.67	3.08(0.53-21.83)	6.67(0.56-74.27)
Pooled results			957	1543	966	3456	1287	2112	1558	4957		1.09(0.97-1.23)	0.90(0.78-1.04)
P for heterogeneity^a^												0.11	0.07
I^2^ test												36.40%	45.30%

OR: odds ratio; CI: confidence interval; HWE: Hardy–Weinberg equilibrium. ^a^*P *< 0.05, it was considered statistically significant.

ERCC1 and ERCC2 genes are two important rate-limiting enzymes, and they play a role in the NER process. ERCC1 is a subunit of the NER complex, and ERCC1 usually interacts with XPA, XPF and RPA and can guides 5’ cleavage activity during the process of NER.^26^^,^^27^ Deficient of ERCC1 expression usually shows a high mutation frequency, an increased level of genomic instability and decreased S-phase-dependent illegitimate chromosome exchange, and it can cause a response adopted by rodent cells to prevent the accumulation of DNA double strand breaks.^28^ ERCC2 is located at chromosome 19q13.3, and this gene possesses both single strand DNA-dependent ATPase and 5’-3’ DNA helicase activities and participates in DNA unwinding during the process of NER pathway.^29^^,^^30^ Variants of ERCC2 gene is reported to have a role in decreasing the helicase activity, causing a lower DNA repair capacity of NER pathway and affecting the risk of cancer.^31^^,^^32^ Therefore, variants of ERCC1 and ERCC2 genes can influence the cellular DNA repair capacity and affect the susceptibility to glioma risk.^11^^,^^19^ Our meta-analysis demonstrated that ERCC1 rs3212986 polymorphism was significantly associated with glioma risk, but ERCC2 rs13181 polymorphism was not correlated with glioma risk. When conducting stratified analysis by ethnicity, ERCC1 rs3212986AA, ERCC2 rs13181 GT and TT genotypes was associated with increased risk of glioma in Chinese population. But there was no significant increased risk in Caucasian population. Our study suggests that ERCC1 rs3212986 and ERCC2 rs13181 showed different effect on the risk of glioma across different populations. 

In our study, we did not detect significant difference between-study heterogeneity of all included case-control studies, and the pooled OR did not alter using the sensitivity analysis. Our study indicates that the results of our study are robust and high stable.

Several limitations in our study should be considered. First, the sample size of cases and controls in the stratified analysis was relatively small, which could reduce the statistical power to find the difference between groups. Second, the studies involving Asian patients were too limited to identify precise gene associations. Third, we could not perform a stratified analysis according to ethnicity because of insufficient data. Future studies may address the effect of the ERCC1 rs3212986 and ERCC2 rs13181 polymorphism on different histological brain tumor subtypes to provide a better understanding of tumor pathogenesis.

In summary, our meta-analysis suggested that ERCC1 rs3212986 and ERCC2 rs13181polymorphism play an important risk factor for brain tumor development in Chinese population, but has no association in Caucasian populations. Since potential biases and confounders could not be completely excluded from this meta-analysis, further studies with larger sample sizes and standardized, unbiased genotyping methods are warranted to confirm our findings.

## Notes

***Contribution of authors***:QKC and ZGW conceived, designed and did statistical analysis & editing of manuscript.JXZ, WDL & YHW did data collection and manuscript writing.
